# T-staging pulmonary oncology from radiological reports using natural language processing: translating into a multi-language setting

**DOI:** 10.1186/s13244-021-01018-1

**Published:** 2021-06-10

**Authors:** J. Martijn Nobel, Sander Puts, Jakob Weiss, Hugo J. W. L. Aerts, Raymond H. Mak, Simon G. F. Robben, André L. A. J. Dekker

**Affiliations:** 1 Department of Radiology and Nuclear Medicine, Maastricht University Medical Center, Postbox 5800, 6202 AZ Maastricht, The Netherlands; 2 School of Health Professions Education, Maastricht University, Maastricht, The Netherlands; 3 Department of Radiation Oncology (MAASTRO), GROW School for Oncology and Developmental Biology, Maastricht University Medical Center, Maastricht, The Netherlands; 4 Artificial Intelligence in Medicine (AIM) Program, Brigham and Women’s Hospital, Harvard Medical School, Boston, USA; 5 Departments of Radiation Oncology and Radiology, Brigham and Women’s Hospital, Dana-Farber Cancer Institute, Harvard Medical School, Boston, USA

**Keywords:** Radiology, Reporting, Natural language processing, Free-text, Classification system

## Abstract

**Background:**

In the era of datafication, it is important that medical data are accurate and structured for multiple applications. Especially data for oncological staging need to be accurate to stage and treat a patient, as well as population-level surveillance and outcome assessment. To support data extraction from free-text radiological reports, Dutch natural language processing (NLP) algorithm was built to quantify T-stage of pulmonary tumors according to the tumor node metastasis (TNM) classification. This structuring tool was translated and validated on English radiological free-text reports. A rule-based algorithm to classify T-stage was trained and validated on, respectively, 200 and 225 English free-text radiological reports from diagnostic computed tomography (CT) obtained for staging of patients with lung cancer. The automated T-stage extracted by the algorithm from the report was compared to manual staging. A graphical user interface was built for training purposes to visualize the results of the algorithm by highlighting the extracted concepts and its modifying context.

**Results:**

Accuracy of the T-stage classifier was 0.89 in the validation set, 0.84 when considering the T-substages, and 0.76 when only considering tumor size. Results were comparable with the Dutch results (respectively, 0.88, 0.89 and 0.79). Most errors were made due to ambiguity issues that could not be solved by the rule-based nature of the algorithm.

**Conclusions:**

NLP can be successfully applied for staging lung cancer from free-text radiological reports in different languages. Focused introduction of machine learning should be introduced in a hybrid approach to improve performance.

**Supplementary Information:**

The online version contains supplementary material available at 10.1186/s13244-021-01018-1.

## Key points

- Oncological staging from free-text radiology reports with NLP is feasible.
- NLP algorithms can be successfully translated and implemented from Dutch to English.

## Background

Radiological reports contain an extensive amount of historical information about the patient and their current disease status over a prolonged period of time [1]. Ideally, information from such reports should be available as structured data that can easily be communicated and reused. Instead, these reports are generally at best semistructured free-text reports, which takes a human reader to interpret. Natural language processing (NLP) techniques provide solutions for the extraction of structured data from unstructured text and has been applied to many healthcare purposes and may help to extract structured information from radiology reports [2].

Specific NLP algorithms already exist to find tumor-specific information in radiological reports to extract, for instance, cancer outcomes [3–6]. Next to extracting tumor endpoints and follow-up from radiological reports, NLP algorithms can also be used to extract tumor staging from free text. An example is a Dutch rule-based NLP algorithm that can extract the T-stage for lung cancer according to the tumor node metastasis (TNM) oncology classification system from the free-text radiological reports of chest computed tomography (CT) scans [7, 8]. Lung cancer is the most common oncological cause of death, with imaging playing a great part in its diagnosis and staging [9]. Therefore, improvements in the reporting and staging process may be valuable. Specifically, it may speed up workflow and enhance the quality and accuracy of the radiological report, as well as communication between health professionals.

The Dutch algorithm analyzes the radiological report and extracts tumor stage with an accuracy score of 0.83–0.87. In addition, this algorithm can also be used for (re)staging historical data, which may be useful, for instance, in cases that have been classified with an older version of the TNM classification system or adjustments with newly available data. An NLP algorithm can therefore function as an important solution to increase the value of the radiological report. Implementation of this NLP algorithm can also act as a method to extract and convert unstructured free-text information into stored structured information from radiological reports. This is important, because structured stored data can be processed more easily than free text for clinical or research purposes [10]. This is of particular interest when realizing that over the past years a shift toward structured reporting has been promoted by the Radiological Society of North America (RSNA) and the European Society of Radiology (ESR). The goal of this is to increase the value of the radiological report and allow for better content datafication [11, 12]. Moreover, the ESR published guidelines for radiologists on reporting and good practice, which highlights the need for better reporting, also promoting the potential of (multilingual) structured reporting [13, 14]. Also, several surveys of radiologists show a global shift toward the use of structured reporting in radiology [15, 16], as many radiologists appreciate the benefits of structured reporting, such as report clarity, communication and data mineability [17, 18]. Although the NLP approach does not use a strict structured reporting format like a point-and-click system, drop-down menu or template to insert structured data elements, it does analyze the old-fashioned free-text report to create structured data during or after the reporting process. Thereby, this NLP algorithm can also be used on old free-text reports to extract T-stage according to the current standards and can help quality assessments of oncological registries.

This algorithm is only capable of processing Dutch staging CT reports and is therefore only proven to be effective in Dutch. With the translation of used regular expressions, it may be possible to translate the algorithm into other languages, like English. In addition, to increase understanding of the algorithm and to utilize its full potential, building a graphical user interface (GUI) might increase the usability and clinical utility of the algorithm. The hypothesis of this study is that the Dutch algorithm can be translated into English to allow for analysis of English free-text radiological reports.

This paper presents the process of translation, implementation and validation of the Dutch pulmonary T-stage algorithm to report written in English with the use of a GUI.

## Methods

### Corpus description

After institutional review board approval at the participating medical center, an existing retrospective lung cancer clinical database of patients treated at the institution was used to search for radiological reports of diagnostic CT or positron emission tomography-computed tomography (PET-CT) scans, performed at initial cancer staging. Inclusion totaled 425 radiological reports of patients with primary pulmonary oncology of which the full report of the staging examination was available. Cases were excluded in case of (1) follow-up and restaging reports (second opinions), (2) cases with two primary tumors or (3) incomplete reports (no full text and/or primary staging report available). The first 200 reports formed a training set; the remainder of the cases composed a validation set (*n* = 225). Tumor and report characteristics of both groups are shown in Table 1.

**Table 1:** Cohort composition of the training and validation sets

	Training (*n* = 200)	Validation (*n* = 225)
*TNM substage*		
T1a	4	6
T1b	27	31
T1c	42	42
T2	6	3
T2a	32	44
T2b	27	23
T3	33	41
T4	29	35
*Report format*		
CT	106	120
PET	77	88
PET-CT	17	17

Included report statistics by T-substage for the training and validation sets

### Determining T-stage

The radiology reports were created using a speech recognition device and contained free text concerning at least the lungs. Three different report formats that could be discerned were all included: a strictly radiological CT report, a PET-CT report in which radiological information was blended with the nuclear diagnostic information and a more structured PET-CT format in which the two types of information were separated in the report. Most reports used subheadings for the body part lung, like *Thorax* or *Chest*. Also, other body parts were described in most of the reports and consisted of different combinations of the following elements: *History*, *Comparison*, *Technique*, *Findings (CT and/or PET-CT)*, *Head*, *Neck*, *Chest*, *Mediastinum*, *Abdomen*, *Pelvis*, *Bones* and *Musculoskeletal*.

Because TNM stage was not separately mentioned in these clinical reports, the T-stage was classified manually retrospectively from the report, according to the AJCC 8^th^ edition TNM classification [7]. The authors agreed on annotation guidelines for proper labeling, and the T-stage was only scored if it was stated as being certain (Additional file 1: Appendix 1 Annotation guidelines). In ambiguous cases, final T-stage was determined after reaching consensus between two authors.

### Modifications for use in English

The training set was used to identify the specific structure and the indentation of the reports. Furthermore, the used subheadings had to be identified in the training set to correctly whitelist or blacklist specific sections of the report. To find proper English synonyms, the Dutch regular expressions, containing all synonyms and variants which are linked to the Systematized NOmenclature of human MEDicine-Computed Tomography (SNOMED-CT) terms [19], were translated and used as a starting point. These Dutch regular expressions were used to build an English Regular Expression (RegEx) per concept, which included the accompanying SNOMED-CT label to assure for proper ontology-based standardized classification. The used synonyms in English and their accompanying RegEx and ontology-based SNOMED-CT terms can be found in Additional file 1: Appendix 2 Concept Synonyms.

### Algorithm structure

**Fig. 1:**
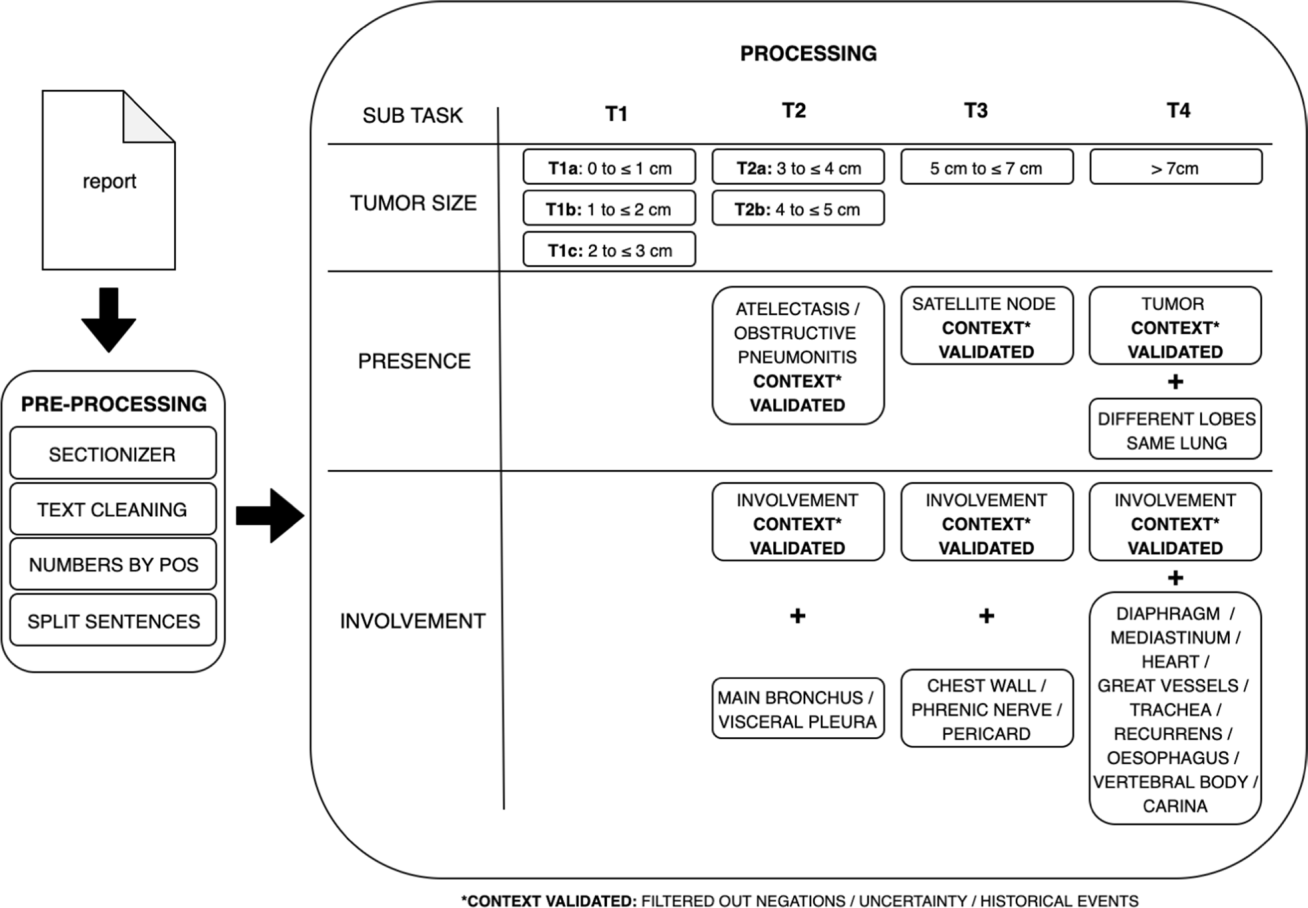
T-stage classifier. Schematic overview of T-stage classification. In the preprocessing step, raw data of the report are prepared for the actual processing. In the processing step, the following subtasks are performed: tumor size extraction, a T-stage presence check of abnormalities and involvement [8]

This study used the same lung cancer T-stage algorithm structure as Dutch language-based algorithm, in which processing was subdivided in a preprocessing and a processing step to consecutively clean and process the radiological report [8]. Three similar items from the T-staging method had to be extracted (*size*, *presence* and *involvement)* before the T-stage classifier was able to stage the full T-stage (Fig. 1). Open-source part-of-speech (POS) tagging, NLP software library spaCy and pyContextNLP were used for number extraction, sentence segmentation and context validation [20, 21]. In addition to the Dutch algorithm, a blacklist had to be added to ignore sentences containing (mass) sizes in organs or body parts other than the lung, as some PET-CT scans covered more than only the thorax.

### Analysis

Analysis of the data was performed in order to assess the separate accuracy scores of the training and validation set for the T-stage (e.g., T1–T4) and the T-substage (e.g., T1a, T1b, T1c). In addition, T-stage, in which only size was used for classification, was calculated and compared with the Dutch results. Recall (i.e., sensitivity), precision (i.e., specificity), and F_1_ measure (i.e., combined metric for precision and recall) for the T-stage classifier have been calculated for all substages in the training and validation set. To further differentiate outcomes, the total number of errors were grouped by category into context, concepts, standardization, complexity ambiguity, preprocessing and reporter.

### Graphical user interface

**Fig. 2:**
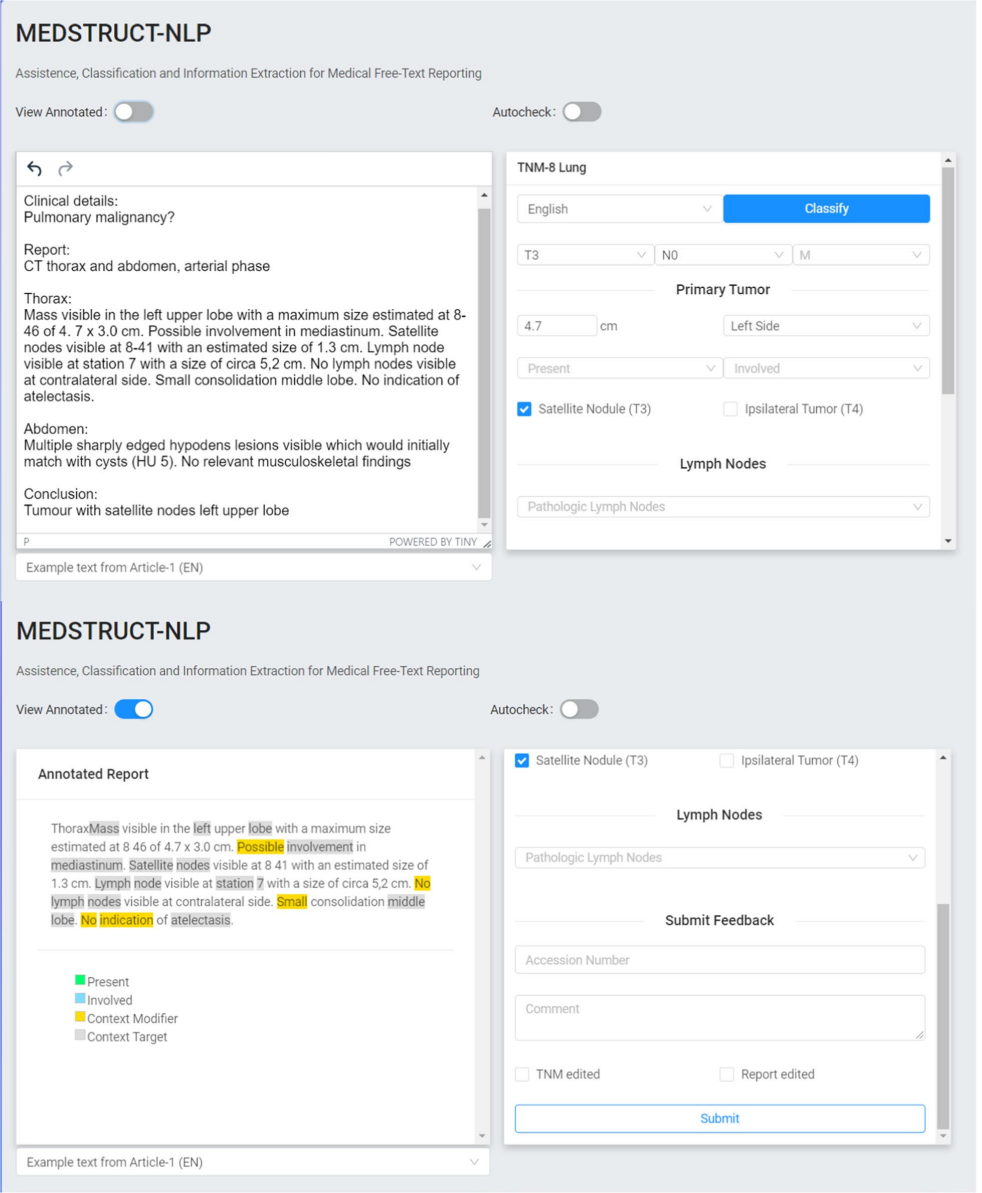
Graphical user interface MedStruct. Two screenshots of the graphical user interface MedStruct with the original report on the left side and its T-stage on the right side, combined with the items size, present and involvement. Also, N (nodal stage) and M (metastatic disease) are mentioned for future use. By using drop-down menus stages can be adjusted (upper). Annotated report at the left side and a feedback form at the right (lower) [22]

**Fig. 3:**
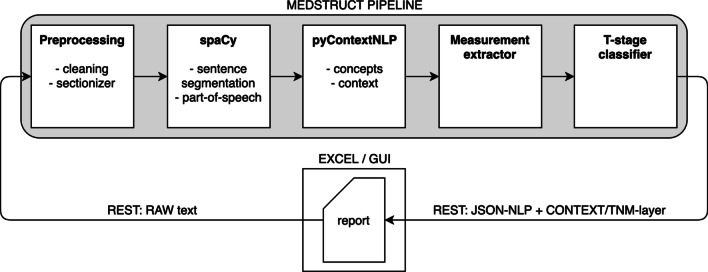
MedStruct pipeline. Schematic overview of the MedStruct pipeline, in which five different microservices are present: preprocessing, spaCy, pyContextNLP, measurement extractor and T-stage classifier. The report can be processed either from an Excel file or direct from the graphical user interface (GUI). All components use an intermediate JavaScript Object Notation (JSON) annotation format to chain the pipeline components and can be consumed over REpresentational State Transfer (REST) or chained using a message broker. The use of a JSON annotation format simplifies reusability of the different components, enables mixing programming languages, prevents for duplicate processing and guarantees token alignment between components. This implementation saves annotations at token level instead of sentence level, which enables precise highlighting of annotations in a GUI. Detected tumor and lymph nodes are stored as objects in a list, allowing for detection of concurrent mentions. Documents can now be processed individually with the same rule-based algorithm

For this study a GUI, called MedStruct, was built to train and visualize the results of the algorithm by highlighting the extracted concepts and its modifying context (Fig. 2) [22]. This was especially useful for finding proper synonyms as well as for analyzing and adjusting errors during training. To enable this GUI, the algorithm has been re-implemented into five reusable NLP pipeline microservices without changing the approach of the algorithm nor the algorithm itself (Fig. 3). The total pipeline now consists of a preprocessing component, spaCy, pyContextNLP, measurement extractor and the T-stage classifier. A web application has been created in which the report can be inserted or edited. The T-stage classification is automatically extracted, and the result is immediately displayed.

The GUI highlights concepts and modifiers found in the report and displays the location, size, presence and involvement items on which the T-stage is based. Items can be adjusted using implemented drop-down menus.

## Results

### Algorithm performance

The manually annotated T-stages and the report formats of the included reports were equally distributed in the training and validation set (Table 1). Only substages T1a and T2 have lower F_1_-scores in both the training and validation sets compared to the other stages. This might be due to the fact that these are underrepresented in this cohort.

The T-stage classifier accuracy was 0.89 for both the training and validation sets, 0.87 and 0.84 when considering the T-substages and 0.78 and 0.76 when only using tumor size for classification (Table 2).

**Table 2:** T-stage classifier accuracy

	English		Dutch	
	Training (*n* = 200)	Validation (*n* = 225)	Training (*n* = 47)	Validation (*n* = 100)
Accuracy T-substage	0.87	0.84	0.79	0.88
Accuracy T-stage	0.89	0.89	0.81	0.89
Tumor size-based T-stage	0.78	0.76	0.70	0.79

Accuracy scores of training set and validation sets in the English cohort and the Dutch cohort. In the Dutch group, the outcomes with the new processing structure are recalculated at the substage level

**Fig. 4:**
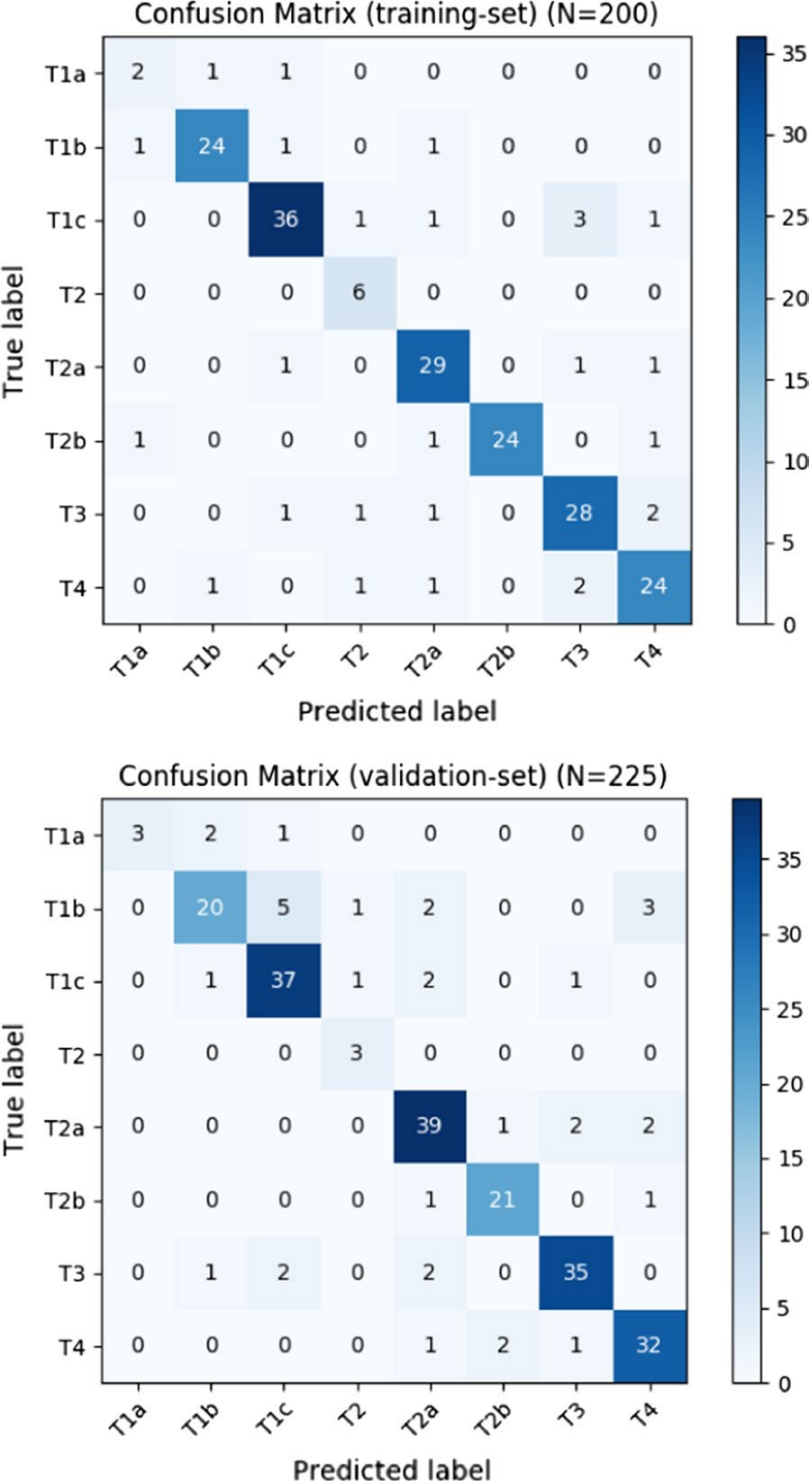
Confusion matrices of the T-stage classification training set (upper) and validation set (lower)

The accuracy rates of the Dutch algorithm are added in the same table, showing the same outcomes in, respectively, the training (*n* = 47) and validation sets (*n* = 100). A confusion matrix is shown in Fig. 4, where actual T-stage *(true label)* is compared with the predicted T-stage *(predicted label).* In addition, the recall (i.e., sensitivity), precision (i.e., specificity), and F_1_ measure (i.e., combined metric for precision and recall) for the T-stage classifier are shown in Table 3.

**Table 3:** Precision, recall and F_1_-scores T-substage

	Precision	Recall	F_1_ score
*Training*			
T1a	0.50	0.50	0.50
T1b	0.92	0.89	0.91
T1c	0.90	0.86	0.88
T2	0.67	1.00	0.80
T2a	0.85	0.91	0.88
T2b	1.00	0.89	0.94
T3	0.82	0.85	0.84
T4	0.83	0.83	0.83
*Validation*			
T1a	1.0	0.50	0.67
T1b	0.83	0.65	0.73
T1c	0.82	0.88	0.85
T2	0.60	1.00	0.75
T2a	0.83	0.89	0.86
T2b	0.88	0.91	0.89
T3	0.90	0.88	0.89
T4	0.84	0.89	0.87

Precision, recall and F_1_-scores T-substage for the training set and validation set

In Table 4, errors made in the training set and validation set have been grouped into specific categories. In total, 27 (13.5%) errors were made in the training set and 35 (15.6%) errors in the validation set. Most errors were scored in the ambiguity category for the training (48.1%) as well as the validation set (51.4%).

**Table 4:** T-stage errors by category

Error group	Error type	Description	Training (*n* = 200)	Validation (*n* = 225)
Data selection	Sectionizer	Detects information in wrong subheadings	1	3
	Missing blacklist synonyms	Falsely matched/falsely not excluded	0	5
Context	Context missing	Context not matched because of missing modifier	1	0
	Context mismatch	Context mismatch, wrong modifier detected	1	3
Concept matching	Measurement extractor	e.g., using abbreviations (e.g., (AP) × (TVR) × (SI))	1	2
	Complexity	T4 multiple lobes	2	1
	Ambiguity	Confusion between node and mass (specific site: hilar)	4	7
		Nonspecific	4	9
	Missing concepts synonyms	Lobulated	1	0
		Cystic	2	0
		Pleural thickening	1	0
		Spinal metastasis	1	0
		Costal involvement	0	1
		Supraclavicular extension	0	1
Reporter	Wrong input	Different sizes for the same tumor, no unit (mm/cm) present, size for tumor and atelectasis	7	2
		Satellite node	1	1
Total errors			27	35

T-stage errors by category for training and validation sets

### Graphical user interface

By using this tool (Fig. 2), the report and the tumor-specific concepts are shown in a structured layout. Items that are present, missing or incorrectly stated can now be visualized. For instance, when tumor size or its unit is missing, size is not mentioned in the user interface and final T-stage will not be extracted properly. To increase its functionality, providing feedback and correcting errors, it is possible to adjust the proposed T-stage by changing the concepts found using drop-down menus overruling the algorithm. In addition, this adjusted report can be saved anonymously and can be used as feedback to further improve the algorithms’ future accuracy. As such, this tool can also function as a corpus builder when reports are being created. A consequence of the language-independent output format of our algorithm is that the Dutch algorithm is also available in the same GUI. The language can be set by clicking on a button.

## Discussion

This study was performed to transfer and externally validate the Dutch rule-based pulmonary tumor T-stage NLP algorithm in an English cohort with the use of a GUI. Accuracy scores in this English study were similar to the scores found in the Dutch cohort. The results confirm that the used strategy according to *size, involvement* and *presence* is viable and can also be implemented in a different language other than Dutch. The approach to find appropriate synonyms according to the Dutch outcomes (i.e., synonyms and found SNOMED-CT terms) was sufficient to get started. Adjusting the synonyms, without changing the algorithm itself, was enough to increase its accuracy. This again shows that the rule-based approach is very promising and can be implemented with a fairly high accuracy. Especially when taking into account that collecting data, and training and validation of the algorithm were done in roughly four weeks.

When looking at the separate F_1_ scores, outcomes are slightly higher in the training set, but still have overall decent scores. The confusion matrices show that this algorithm tends to slightly overstage lower T-stages (up to T2a) and slightly understage the higher T-stages (from T2b onwards). This can be partly explained by the fact that it is more plausible to overstage a lower T-stage and understage a higher T-stage. However, as described in the following sections, this is most likely the result of difficulties experienced with the *overall reporting differences* and can be further explained with highlighting the *errors made by category, improving the algorithm* and the use of a *graphical user interface*.

### Overall reporting differences

One of the most important things was to find differences in reporting manner between the Dutch and English setting. Therefore, it was necessary to analyze the reports on a fundamental basis to find differences in reporting manner and used vocabulary in addition to the local used subheadings and layout. Because this cohort also used PET-CT scans in addition to CT scans, subheadings had to be added and the processing format had to be adjusted.

When looking at the reporting manner and the vocabulary used, the description of lymph node locations was found to be different in English as they are described in words (e.g., subcarinal) and not by numbers (e.g., level 7) as commonly done in Dutch reports. Another important finding was that the word ‘involvement’ and its conjugations (‘involv’-ing) were not exactly interchangeable, because involving has a more ambiguous meaning in English than the Dutch word for involvement (‘ingroei’—extension), which is very specific.

Furthermore, Dutch reports mention involvement when involvement is certain or suspected with a high level of certainty. Possible but less certain involvement is commonly not mentioned. The included English reports use more frequently terms to describe possible invasion without stating the exact certainty of the invasion with words such as ‘extending towards,’ ‘abutting’ or ‘in close relation to the tumor’. As the algorithm was trained on matching the specific concept for invasion and the invaded concept, outcomes were more often falsely matched, in turn leading to false-positive results.

### Errors made by category

#### Data selection

In this category especially the blacklist in the validation set was not sufficient enough, which resulted in five entities that were falsely classified as tumor, but were benign lesions (for instance a benign kidney cyst with a size). As a consequence, these benign entities and its sizes were falsely seen as a tumor, resulting in an overestimation of the actual tumor size.

#### Context matching

Errors made in this category were due to a mismatch between the concept and the context. This happened for instance when a report lacked tumor dimensions, but instead was called ‘large’. Because tumor size is needed for the algorithm to appreciate something as a mass, this mass was missed. Another difficulty was to find sufficient synonyms for the ambiguous term atelectasis, which can be referring to either a post-obstructive atelectasis, which is a concept for the item *presence* in T2 tumors, or a regular seen non-tumoral gravity-related atelectasis. Specific atelectasis-related adjectives (basal, bilateral, subsegmental, etc.) were used to exclude gravity-related atelectasis, as was done in the Dutch approach. However, this could not prevent some mismatches and overstaging of T1 tumors.

#### Concept matching

Most errors are related to this category with several subcategories, of which ambiguity is the largest contributor. Errors in this category were mainly due to difficulties in differentiating a lymph node from a tumor mass and difficulties in finding its proper location. This is especially true for the hilar region. For instance, a lymph node can be described as ‘a (lymphnodal) subcarinal mass’ and a tumor as ‘a (peri)hilar node,’ making its exact location, and whether this is a primary tumor, less clear. Inserting more specific synonyms for involvement (i.e., involvement in(to)) and specific terms for lymph node and mass location (i.e., subcarinal lymph node) increased accuracy, but could not be solve this problem entirely. This was in Dutch reports a lesser problem because lymph node levels are mostly mentioned by level number.

The error type missing/misuse concept synonyms is of particular interest because it shows difficulties caused by the rule-based algorithm approach best. One error in this subcategory was made because there was a size at an involvement concept (visceral pleura) that therefore could not be blacklisted (e.g., ‘pleural thickening of 8,6 cm’). Also the opposite errors existed in cases, in which a cystic pulmonary tumor was missed because the word cyst was blacklisted to not falsely match a renal cyst. In addition, it was not possible to differentiate osseous destruction caused by the primary tumor from destruction caused by a vertebral metastasis. The complexity subcategory and errors made by the measurement extractor have similar difficulties in which it is difficult to match a different tumor in the same lobe of the ipsilateral lung or match a tumor size when the size is written in an uncommon format.

#### Reporter

This category included errors which can be explained by stating wrong tumor size, or mentioning it twice, or incorrectly reporting the presence of a satellite nodule. As the algorithm demands a unit for every size with its size is correlated to the stated lobe, it is possible that a tumor without a unit is missed, a tumor with two different sizes is overestimated and a satellite nodule in a different lobe is missed. As such, correctly stated input is of great importance.

### Improving the algorithm

The described rule-based algorithm is promising, but this approach is a trade-off between missing a lung tumor and finding a false mass. The rule-based nature of the use of the sectionizer and regular expressions is not extensive enough to exclude the ambiguities, nor to find sections when those were not present in the training set. Furthermore, the context analysis does not search for dependency relations, resulting in mismatches between concept and context. In addition, the rule-based approach does not seem extensive enough, as the T-stage based on only tumor size has an accuracy of, respectively, 0.78 and 0.76 in the training and validation sets. The additional set of rules improves the outcome only by 0.08–0.09.

Although NLP can be successfully applied in free-text reports, its accuracy will benefit from increasing levels of structure and standardization in the report. In addition, machine learning is thought to increase the accuracy score by finding more related synonyms based on a larger amount of data. This can be achieved by using, for instance, word embeddings. This allows for more extensive analysis of the context, because specific concepts are often embedded by the same set of modifiers.

Although machine learning may be a promising addition, it requires much more annotated data for training purposes. Availability of these large amounts of specific data is sometimes an issue, especially at the beginning of a new measurement method or a new edition of the TNM staging. In addition, extracting and labeling large amounts of data are expensive and time-consuming. Therefore, it is important to learn from this baseline study and explore where exactly implementation of machine learning or deep learning methods could increase outcomes. Focusing on finding accurate synonyms (e.g., gravity-dependent atelectasis/non-oncological atelectasis), distinguishing tumor from lymph node and accurate matching of contextual information to the right concepts might be a way to improve the algorithm. This hybrid approach could increase outcomes more efficiently, without the need to annotate a vast amount of data. This could result in lower costs and speeds up the availability of these algorithms. In addition, less *specific* data can be used to train the algorithm because only the experienced difficulties need to be trained. For instance, non-oncological atelectasis is also mentioned in non-oncological CT or PET-CT scans.

### Clinical significance and future perspectives

Future work should focus on improving the algorithm, but research can also be aimed at how such algorithms can help with restaging tumor classifications across staging editions or how a classification GUI can be implemented in clinical practice.

In this study, the GUI is only used for finding, analyzing and adjusting errors during training. However, this tool can also be implemented for (live) staging during the reporting process. When connected to the directory in which reports are made, (live) staging aids the reporter in increasing accuracy, completeness and quality of the report by making sure that specific concepts are mentioned in the free-text report by looking at the (already filled in) structured format. The GUI can notify the reporter with a pop-up screen that pivotal information is missing. In this study, 8 and 3 reporter related errors were found in, respectively, the training and validation set. These could be prevented when information was checked before finishing the report. The use of this algorithm with the GUI could have increased the report accuracy (i.e., quality of the report) by 1.5–4.0%. As such, the GUI might lead to better reports and perhaps also nudge the radiologist to more structured and standardized reporting as they see the direct effect of that in the GUI.

Moreover, the potential of these types of algorithms will be further enhanced when they are used in less difficult settings, like automatic extraction of the TIRADS (Thyroid Imaging Reporting And Database System) classification of thyroid nodules as described in thyroid ultrasounds. Automatically stating the Bosniak classification on CT scans used to describe cystic renal masses may be another example. When we can also combine artificial intelligence (AI)-based automated image extraction information tools (e.g., tumor size extractor), it might be possible to prefill the radiological report and assist the reporter and the algorithm further.

A different opportunity of NLP is to extract certain endpoints, such as the presence of a specific disease or important or incidental findings. This can be used to (semi)automatically warn the referring specialist or plan a follow-up appointment.

As such, applying these algorithms in clinical practice can be complementary to structured reporting in radiology. It automatically checks the free-text report for specific items and converts these items into a structured format, without extensively changing or interfering the way of reporting. This is especially of importance in times of datafication and increased need for data standardization as promoted by the ESR and RSNA. It shows that also NLP or rule-based algorithms can reinforce the radiologist and their reports, further supporting the reporting process.

## Limitations

A limitation of using a rule-based approach building this T-stage algorithm is that specific boundaries had to be determined if those were not specified by the TNM. For instance, it was necessary to specify the size of the node in the ipsilateral side of the main tumor in a different lobe for T4 stage (> 1 cm) and the size for a different tumor in a different lobe (> 1 cm).

Another limitation was that we had to determine the strictness of the algorithm and more specifically on concepts such as involvement or presence. It is debatable whether only obvious invasion should be accounted for an involved concept or whether terms like ‘likely’ or ‘probably’ should be added to the invaded concepts. However, the presented rule-based algorithm can be configured. Furthermore, the obtained T-stage by this algorithm is a radiological T-stage. This may be different from the final T-stage, which generally also requires additional clinical information.

Lastly, the T-stage scoring process was done by one author (J.M.N.). In case of uncertainty and/or ambiguity, a second author (J.W.) was consulted, after which consensus was reached between two authors. Although future validation studies should also look at aspects of interrater variability, the primary goal of the current study is to explore whether the Dutch algorithm could be useful when translated into English.

## Conclusions

NLP is a promising tool that can be used in extracting specific information from radiological reports concerning T-stage in pulmonary oncology. The used Dutch algorithm could be successfully translated and validated in an English dataset, and this will likely be feasible for other languages as well. Focused implementation of more machine learning strategies and the use of a graphical user interface should lead to higher accuracy, as an effect of better report quality.

## Supplementary Information

**Additional file 1.** **Appendix 1**. Annotation guidelines. **Appendix 2**. Concept Synonyms.

## Data Availability

The data that support the findings of this study are obtained from the Departments of Radiation Oncology and Radiology, Brigham and Women’s Hospital/Dana-Farber Cancer Institute (Boston, United States of America), but restrictions apply to the availability of these data, which were used under license for the current study, and so are not publicly available. Data are, however, available from the authors upon reasonable request and with permission of the above-mentioned party.

## Abbreviations

AI: Artificial intelligence
CT: Computed tomography
ESR: European Society of Radiology
GUI: Graphical user interface
JSON: JavaScript Object Notation
NLP: Natural language processing
PET-CT: Positron emission tomography-computed tomography
POS tagging: Part-of-speech tagging
RegEx: Regular expression
REST: REpresentational State Transfer
RSNA: Radiological Society of North America
SNOMED-CT: Systematized NOmenclature of human MEDicine-Computed Tomography
TIRADS: Thyroid Imaging Reporting And Database System
TNM: Tumor node metastasis
