# Supplementary material for: T-staging pulmonary oncology from radiological reports using natural language processing: translating into a multi-language setting
Source: Insights Imaging. 2021 Jun 10;12:77. doi: 10.1186/s13244-021-01018-1 (PMC8192634; doi:10.1186/s13244-021-01018-1)
Supplement: Supplementary file 1 — Additional file 1. Appendix 1. Annotation guidelines. Appendix 2. Concept Synonyms. [file 13244_2021_1018_MOESM1_ESM.docx]

**ELECTRONIC SUPPLEMENTARY MATERIAL**

**Appendix 1 Annotation guidelines**

Stated as being certain

Secondary tumor ipsilateral: size > 1,0 cm

Atelectasis by tumor

Satellite nodules only when in the same lobe

**Appendix 2 Concept synonyms**

*Regular expressions used for classification concepts tumor and involvement, corresponding SNOMED-CT concepts.*

General

| concept | regular expression | SNOMED CT concept |
| --- | --- | --- |
| tumor | '(tumor\|tumour\|carcino\|malign)' | 108369006 \| Neoplasm (morphologic abnormality) |
| involvement | 'affecting\|attacking\|injuring\|destruct\|ingrowth\|growth\|(extension\|extends\|extending\|expansion)[ ]*(in\|into)\|(involvement\|infiltration)[ ]*of\|involve\|invading in\|invas\|invades' | 248448006 \| Involved (qualifier value) |
| lymph nodes | 'lymph\|lymph.*node' | 59441001 \| Structure of lymph node (body structure) \| |

T2 Presence

| concept | regular expression | SNOMED CT concept |
| --- | --- | --- |
| main_bronchus | '(central\|main\|first).*bronch' | 102297006 \| Main bronchus structure (body structure) |
| visceral_pleura | 'pleura' | 81623005 \| Visceral pleura structure (body structure) |

T2 Involvement

| concept | regular expression | SNOMED CT concept |
| --- | --- | --- |
| atelectasis | 'atelect\|collapse' | 46621007 \| Atelectasis (disorder) |
| obstructive_pneumonitis | 'obstructive pneumoni\|infect.*chang\|obstructive pneumonitis\|obstructive infectious disease\|obstructive pneumonia' | 205237003 \| Pneumonitis (disorder) |

T3 Involvement

| concept | regular expression | SNOMED CT concept |
| --- | --- | --- |
| chest_wall | 'chest.*wall\|thorax.*wall\|rib\|costa’ | 78904004 \| Chest wall structure (body structure) |
| nervus_phrenicus | 'nervus.*(phrenicus\|frenicus)\|phrenic nerve\|nervus phrenicus' | 50230006 \| Structure of phrenic nerve (body structure) |
| parietale_pericard | 'pericard' | 76848001 \| Pericardial structure (body structure) |

T3 Presence

| concept | regular expression | SNOMED CT concept |
| --- | --- | --- |
| satellite_nodule | 'satellite nodule\|satellite nod\|satellite lesion' | 396408009 \| Specimen involvement by satellite nodule(s) present (finding) |

T4 Involvement

| concept | regular expression | SNOMED CT concept |
| --- | --- | --- |
| diafragm | 'diaphragm' | 5798000 \| Diaphragm structure (body structure) |
| mediastinum | 'mediast\|mediastinum\|mediastinal fat' | 72410000 \| Mediastinal structure (body structure) |
| heart | 'heart\|cor\|cardial' | 80891009 \| Heart structure (body structure) |
| great vessels | 'great vessel\|great vessels\|central vessels\|central vessel\|aorta\|vena cava\|VCS\|main pulmonary artery\|main pulmonary vein' | 3711007 \| Structure of great blood vessel (organ) (body structure) |
| trachea | 'windpipe\|\btrachea\b\|\btracheal\b' | 44567001 \| Tracheal structure (body structure) |
| recurrent_laryngeal_nerve | 'recurrent laryngeal nerve\|laryngeal nerve\| nervus laryngeus recurrens' | 731050007 \| Entire recurrent laryngeal nerve (body structure) |
| oesophagus | 'oesophagus\|esophagus' | 32849002 \| Esophageal structure (body structure) |
| Vertebral body | 'vertebral body\|vertebra\|spine\|spinal' | 3572006 \| Structure of body of vertebra (body structure) |
| carina | '\bcarina\b\|\bcarinal\b' | 28700002 \| Structure of carina of trachea (body structure) |

T4 Tumor in different lobes

| concept | regular expression | SNOMED CT concept |
| --- | --- | --- |
| superior_lobe_left | 'superior left lobe\|superior lobe left\|upper left lobe\|left upper lobe\|upper left lobe\|apical left lung\|apical lung left\|upper lobe of left\|\bLUL\b\|\bSLL\b' | 44714003 \| Structure of upper lobe of left lung (body structure) |
| middle_lobe | 'middle lobe\|center lobe\|lobus intermedius\|\bML\b\|\bRML\b' | 72481006 \| Structure of middle lobe of right lung (body structure) |
| inferior_lobe_right | 'inferior right lobe\|inferior lobe right\|lower right lobe\|right lower lobe\|lower lobe right\|basal lung right\|basal right lung\|\bRLL\b\|\bILR\b' | 266005 \| Structure of lower lobe of right lung (body structure) |
| inferior_lobe_left | 'inferior left lobe\|inferior lobe left\|lower left lobe\|left lower lobe\|lower lobe left\|basal lung left\|basal left lobe\|\bLLL\b\|\bILL\b' | 41224006 \| Structure of lower lobe of left lung (body structure) |
